# Reviews

**Published:** 1862-03

**Authors:** 


### REVIEWS.

*A System of Surgery; Pathological, Diagnostic, Therapeutic and Operative, by* SAMUEL D. GROSS, M. D., *Professor of Surgery in the Jefferson Medical College of Philadelphia; Surgeon to the Philadelphia Hospital; Member of the Imperial Royal Medical Society of Vienna, etc., etc. Illustrated by twelve hundred and twenty-seven engravings. Second edition, much enlarged and carefully revised, in two volumes. Philadelphia:* BLANCHARD & LEA, 1862.

That the first edition of this work has been so soon exhausted, shows most conclusively that it is appreciated by the profession; and also indicates that there is no necessity for an extended notice, since most of those who practice surgery are familiar with its pages.

In this second edition every chapter has been carefully revised, a large amount of new matter introduced, and nearly three hundred illustrations added. The subject of gun-shot wounds has received more than ordinary attention, since it is invested at this time with such a fearful interest in this country. The general arrangement of the work is the same as in the first edition. The new matter which has been introduced is very valuable since it is essentially of a practical nature. This work constitutes a systematic and comprehensive treatise on the science and practice of Surgery considered in its broadest sense, and will serve the practitioner as a faithful and available guide in his daily routine, and constitute a standard authority of the highest value. It embraces the whole domain of surgery, bestowing proper attention to every subject in the great family of external diseases and accidents. There is no topic properly appertaining to Surgery which has not been considered in greater or less extent in these volumes. It may be regarded as embodying the result of the most extensive personal experience and ripe scholarship, aided by the labors of cotemporaries both among systematic writers and contributors to the periodical press of this and other countries, and constitutes the most comprehensive and valuable treatise upon Surgery, ever yet published. The typographical and mechanical part of this book is in the very highest style of Messrs. *Blanchard & Lea,* who have placed the profession under lasting obligations for the durable and substantial form in which their most valuable medical books have been issued.

---

*Notes on the Surgery of the War of the Crimea, with remarks on the treatment of gun-shot wounds, by* GEORGE H. B. MACLEOD, M. D., F. R. C. S.; *formerly Surgeon to the Civil Hospital at Smyrna, and to the General Hospital in Camp before Sebastopol; Lecturer on Military Surgery in Anderson University, Glasgow, etc. Philadelphia:* J. B. LIPPINCOTT & Co., 1862. *London:* JOHN CHURCHILL—$1.50.

These notes are upon the following subjects: History and physical characters of the Crimea—Its Climate and Geology—The changes of the seasons during the occupation of the Allies—The steppe lands of the Interior—Vegetation and resources of the country—The Natives and their diseases—Drainage of the Camp—Water Supply—Latrines—Fort—Cooking—Fuel—Clothing—Housing—Duty—Effects of all these combined upon the health and disease of the soldiers—Hospitals—Distribution of the sick—Male and Female—Nursing—Transport.

Campaign in Bulgaria, and its effects upon the subsequent health of the troops—The Diseases which appeared there, as well as afterwards in the camp before Sebastopol—Distinction between Surgery as practiced in the army and in civil life—Soldiers as patients and the character of the injuries to which they are liable—Wounds and injuries seen during the late war.

Peculiarities of gun-shot wounds and their general treatment—Chloroform in the Crimea—Primary and Secondary hemorrhage from gun-shot wounds—Zetanus Gangrene—Erysipelas—Frost-bite—Injuries of the head—Wounds of the face and chest—Gun-shot wounds of the Abdomen and Bladder—Compound fracture of the Extremities—Gun-shot wounds of joints—Excision of joints—Amputation—to which is added an Apendix.

This book is written in a very entertaining and instructive style; and at this time the subjects presented must be of great importance to the practical surgeon, since it embodies the results of surgical practice in the most extensive field ever yet opened for observation in Military Surgery. For sale by BREED, BUTLER & Co., No. 188 Main Street, Buffalo.

---

*Braithwaite's Retrospect—The Retrospect of Practical Medicine and Surgery, being a half-yearly Journal, containing a retrospective view of every discovery and practical improvement in the Medical Sciences; Edited by* W. BRAITHWAITE, M. D., *Lecturer on Obstetric Medicine at*

*the Leed's School of Medicine, etc., and* J. Braithwaite, M. D. *American edition. New York:* W. A. Townsend, 39 *Walker street*

This invaluable compendium, which was commenced in 1840, is issued simultaneously with the London edition, and appears regularly in January and July of each year. The peculiar excellence of the *Retrospect* consists in the fact that it embodies in a confined space, the cream of the medical periodicals—preserving the essentially practical articles of discovery and improvement. It constitutes a condensed register of medical facts and observations for the past year, and presents a complete retrospect of the current medical literature of the times, including the transactions of the different Societies and Associations in Europe and America, preserved in as condensed form as possible, and generally in the words of the respective authors. This admirable *digest* enjoys, throughout the world, the highest fame in its department and deserves the universal patronage of the profession.

---

*Report to the Secretary of War of the Operations of the Sanitary Commission upon the Sanitary condition of the Volunteer Army; its Medical Staff, Hospitals and Hospital Supplies, together with accompanying papers from Associate Secretary,* J. S. Newberry, M. D.

"The Commission, having enrolled among its associate members, many distinguished members of the medical profession throughout the loyal States, has thought it fairly within the scope of its duties to invite them to aid in the protection of the Army against disease by the preparation of papers intended to embody in a brief compass, the latest results of Medical and Surgical Science, in regard to various special points of great practical importance, as to which some of our volunteer surgeons necessar ly inexperienced in their new field of army medicine, surgery and hygiene, and without access to libraries, may need information and advice. The duty of compiling these papers has been confided to leading members of the profession in some of the larger cities; and papers upon the *Value of Vaccination in Armies—Pneumonia—Use of Quinine as a Prophylactic against malarious diseases—Continued Fevers—Amputations—Amputation through the foot and at the ankle-joint*, and some others, are now completed, which the Commission propose to place in the hands of every member of the Medical Staff."

We have not space to speak of these papers separately; they are written by eminent men in the profession, and embody the most approved modes of practice. We suppose these papers are designed only for the oneeighth, since the Commission inform us that they "find about seven-eights of the surgeons and their assistants *fairly* qualified for their duties." This commission may have done some good; it probably has accomplished some of the objects of its organization. Large sums of money have been poured into the treasury of the Commission, the "outgushings of affection and patriotism," yet only a little over one-eighth of the money contributed has been expended for the direct benefit of the soldier.

